# Computer-Aided Diagnosis Research of a Lung Tumor Based on a Deep Convolutional Neural Network and Global Features

**DOI:** 10.1155/2021/5513746

**Published:** 2021-02-28

**Authors:** Huiling Lu

**Affiliations:** ^1^School of Science, Ningxia Medical University, Yinchuan 750004, China; ^2^Key Laboratory of Images & Graphics Intelligent Processing of State Ethnic Affairs Commission, North Minzu University, Yinchuan 750021, China

## Abstract

Based on the better generalization ability and the feature learning ability of the deep convolutional neural network, it is very significant to use the DCNN on the computer-aided diagnosis of a lung tumor. Firstly, a deep convolutional neural network was constructed according to the fuzzy characteristics and the complexity of lung CT images. Secondly, the relation between model parameters (iterations, different resolution) and recognition rate is discussed. Thirdly, the effects of different model structures for the identification of a lung tumor were analyzed by changing convolution kernel size, feature dimension, and depth of the network. Fourthly, the different optimization methods on how to influence the DCNN performance were discussed from three aspects containing pooling methods (maximum pooling and mean pooling), activation function (sigmoid and ReLU), and training algorithm (batch gradient descent and gradient descent with momentum). Finally, the experimental results verified the feasibility of DCNN used on computer-aided diagnosis of lung tumors, and it can achieve a good recognition rate when selecting the appropriate model parameters and model structure and using the method of gradient descent with momentum.

## 1. Introduction

Lung cancer is regarded as one of the malignant tumors with high morbidity and mortality, which is a serious threat to human health and life, and it is very difficult for lung cancer patients to be discovered and diagnosed because there are no apparent symptom and typical imaging performance [1]; therefore, early detection and diagnosis are essential for lung cancer patients. In the early diagnosis of lung cancer, multilayer spiral CT (MSCT) can show the transverse, vertical, and coronal planes of the lesion area clearly by a reconstruction technique. In the interim diagnosis, MSCT jointed surface shadowing and multiple planar reconstruction can clearly show the location of the tumor, internal structure, edge features, blood supply, extent of surrounding tissue invasion, and surrounding tissue changes; it gets high accuracy [2]. Hence, the CT image provides an important reference for the diagnosis and identification of lung cancer. Aimed at the massive medical image data, it can reduce the doctors' workload, improve the recognition rate, and reduce the misdiagnosis rate and missed diagnosis rate with the help of computer-aided diagnosis (CAD).

Deep learning [3] as a new field of machine learning analyses and interprets data through simulating a human brain. In particular, a convolutional neural network with the unique deep structure can learn the complex mapping between input and output effectively. At present, the design of the DCNN model is mainly focused on the model parameters, the activation function, the size of the receptive field, the designation of the pooling layer, and so on. Based on the classical model LeNet-5 structure, Chen [4] constructed several different convolutional neural networks by adjusting the number of parameters and the interlayer connection mode, and they were used on optical digital recognition. Ma et al. [5] simplified network structure by removing the third convolutional layer of LeNet-5 and replacing the softmax classifier with an SVM classifier. Hinton constructed a CNN with five convolutional layers, and it achieved good results when used on ImageNet data set [6] in 2012. Gao [7] used whitening pretreatment and stochastic pooling based on traditional CNN; it improved the network generalization ability for military image classification by this method. Zhang et al. [8] constructed a deep convolutional neural network (DCNN) with seven layers for the vehicle type identification; the recognition rate reached 96.8% based on comparative experiment with different model parameters. Guo [9] constructed a DCNN used on hand-printed character recognition; the experimental results show that the receptive field size has a significant influence on the number of model parameters but has little effect on the recognition rate and the running time is in a reverse trend. He and Sun [10] discussed how to balance the number of layers, the number of feature maps, and the size of the convolution kernel in the limited training time and computational complexity; they showed that the recognition performance of CNN with small convolution kernel and deep layers is better than that with large convolution kernel and shallow layers. Gunavathi et al. [11] give a review on convolutional neural network-based deep learning methods in gene expression data for disease diagnosis. Zhou et al. [12] propose a lung tumor Computer-aided diagnosis model in chest CT image based on DenseNet-NSCR (Non-negative, Sparse and collaborative representation classification of DenseNet) in this paper; the result shows that the DenseNet+NSCR model has better robustness and generalization capabilities compared with AlexNet+SVM, AlexNet+SRC, AlexNet+NSCR, GoogleNet+SVM, GoogleNet+SRC, GoogleNet+NSCR, DenseNet-201+SVM, and DenseNet-201+SRC. Zhou et al. [13] use AlexNet, GoogleNet, and ResNet to realize the ensemble deep learning model for novel COVID-19 on CT images. A novel method for stock trend prediction uses a graph convolutional feature-based convolutional neural network (GC-CNN) model, in which both stock market information and individual stock information are considered in Chen et al. [14]. The deep convolutional neural network (DCNN) [15] can automatically extract the high-level features of the image and express the image effectively, and the data is mapped into a new space by the linear or nonlinear transformation of the input data; by this way, the essential feature of an image can be extracted effectively and stably. However, it is necessary to optimize the DCNN for a specific research object and application field. In this paper, the deep convolutional neural network was proposed to identify a lung tumor based on global features of the CT image; on the basis of the original DCNN, the effects of different model parameters, model structure, and optimization algorithm on the recognition performance are discussed in order to validate the feasibility of DCNN used on computer-aided diagnosis of lung tumors and provide a reference for computer-aided diagnosis of a lung tumor.

## 2. Method and Material

### 2.1. DCNN Model Structure

The deep convolutional neural network model is a simulation of simple and complex cell function in the visual cortex, and it extracted features through the alternate convolutional layer and pooling layer and combined with the corresponding classifier to realize image recognition.

#### 2.1.1. Convolutional Layer

Each convolutional layer [16] is composed of multiple feature maps, the neurons in each feature map are connected to the neurons in the input layer or the pooling layer, and the neuron in the same feature map is connected with the neuron in the corresponding receptive field sharing weight; each output feature map can be combined with multiple feature maps:

(1) $$\begin{array}{c} x_{j}^{l}=f\left(\underset{i\in M_{j}}{\sum }x_{i}^{l-1}*k_{ij}^{l}+b_{i}^{j}\right), \end{array}$$

where *x*_*j*_^*l*^ is the output map of channel *j* in convolutional layer *l*, *k*_*ij*_^*l*^ represents the convolution kernel matrix, and *b*_*i*_^*j*^ is the bias; the different convolution kernel weights have different convolution operations.

#### 2.1.2. Pooling Layer

Each pooling layer [17] is also composed of multiple feature maps, the number of feature maps are the same as the number of feature maps in the convolutional layer, and the neuron values are calculated by the maximum or average pooling. The pooling formula is as follows:

(2) $$\begin{array}{c} x_{j}^{l}=f\left(\beta_{j}^{l}*\text{down}\left(x_{i}^{l-1}\right)+b_{i}^{j}\right), \end{array}$$

where down(·) represents the pooling function, and each output image has its own multiply bias *β* and additive bias *b*.

#### 2.1.3. Full Connection Layer

In the full connection layer, the feature maps of all the two dimensional images are connected to one dimension features as the input of the full connection network, and the output of the full connection layer is obtained by the weighted summation of inputs and calculating the response of the activation function:

(3) $$\begin{array}{c} x^{l}=f\left(w^{l}x^{l-1}+b^{l}\right) \end{array}$$

where *w*^*l*^ is the weight coefficient of the connected network, *x*^*l*−1^ represents feature maps, and *b*^*l*^ is the bias of the full connection layer.

### 2.2. Training Method of DCNN

The DCNN training process mainly used the backpropagation algorithm, that is, training data input, activation values of each neuron calculation, then error calculation, gradient calculation of each weight and bias, and weight and deviation adjustment.

#### 2.2.1. Gradient Calculation of the Full Connection Layer

For the full connection layer of DCNN, the BP [18] is used to calculate the partial derivative that error function acts on weight bias. Assuming a multiclassification problem contains *N* training samples and *C* types, the formula of error function is *E*^*N*^ = 1/2∑_*n*=1_^*N*^∑_*k*−1_^*c*^(*t*_*k*_^*n*^ − *y*_*k*_^*n*^)^2^, where *t*_*k*_^*n*^ represents a class label corresponding to the first *k* dimension between *n* samples and *y*_*k*_^*n*^ represents the predicted output value corresponding to the first *k* dimension between *n* samples.

The “error” of the backpropagation network is regarded as the “sensitivity” of each neural unit to the deviation (residual), and the partial derivative of error with respect to network parameters is defined as follows:

(4) $$\begin{array}{c} \frac{\partial E}{\partial b}=\frac{\partial E}{\partial u}\frac{\partial u}{\partial b}=\delta , \end{array}$$

in the formula *∂u*/*∂b* = 1.

The sensitivity of output layer neurons was calculated by the following formula:

(5) $$\begin{array}{c} \delta^{L}=f^{\prime }\left(u^{L}\right)\circ \left(y^{n}-t^{n}\right), \end{array}$$

where “∘” expresses the dot product; that is, the corresponding elements in the matrix were multiplied.

The sensitivity calculation formula of the full connection layer is *δ*^*l*^ = (*W*^*l*+1^)^*T*^*δ*^*l*+1^∘*f*′(*u*^*l*^).

The update rule for the weights of neurons is to multiply the input of the neuron with the triangle of the neuron. It is the inner product of the input vector and the residual vector by the vector representation:

(6) $$\begin{array}{c} \frac{\partial E}{\partial W^{l}}=x^{l-1}{\left(\delta^{l}\right)}^{T},\\ \Delta W^{l}=-\eta \frac{\partial E}{\partial W^{l}}. \end{array}$$

Usually, each weight *w*_*ij*_ has a corresponding *η*_*ij*_ differently.

#### 2.2.2. Gradient Calculation of the Convolutional Layer

Each convolutional layer *l* of CNN is connected with the pooling layer *l* + 1; in the process of backpropagation, we need to sum up all the residuals in the layer *l* + 1 corresponding to the neuron and calculate the residuals of neurons in the layer *l*; then, these residuals are multiplied by the corresponding weights and multiplied by the function of the current neuron. Calculate sensitivity by chain derivation:

(7) $$\begin{array}{c} \delta_{j}^{l}=\beta_{j}^{l+1}\left(f^{\prime }\left(u_{j}^{l}\right)\circ \text{up}\left(\delta_{j}^{l+1}\right)\right), \end{array}$$

where up(·) represents upsampling.

The gradient formula of bias *b* by *δ*_*j*_^*l*^ is *∂E*/*∂b*_*j*_ = ∑_*u*,*v*_(*δ*_*j*_^*l*^)_*uv*_.

The gradient formula of bias *k* by using MATLAB is

(8) $$\begin{array}{c} \frac{\partial E}{\partial k_{ij}^{l}}=\text{rot}180\left(\text{conv}2\left(x_{i}^{l-1},\text{rot}180\left(\delta_{j}^{l}\right),{}^{\prime }\text{vali}{\text{d}}^{\prime }\right)\right). \end{array}$$

#### 2.2.3. Gradient Calculation of the Pooling Layer

In the backpropagation process of the pooling layer, the residual graph is first calculated, and then, the two learning parameters with *β* and *b* are updated.

*δ* can be calculated by MATLAB:

(9) $$\begin{array}{c} \delta_{j}^{l}=f^{\prime }\left(u_{j}^{l}\right)\circ \text{conv}2\left(\delta_{j}^{l+1},\text{rot}180\left(k_{j}^{l+1}\right),{}^{\prime }\text{ful}{\text{l}}^{\prime }\right). \end{array}$$

The gradient of the additive bias *b* is the sum of the elements in the residual graph:

(10) $$\begin{array}{c} \frac{\partial E}{\partial b_{j}}=\underset{u,v}{\sum }{\left(\delta_{j}^{l}\right)}_{uv}. \end{array}$$

The gradient of the multiplicative bias *β* is *∂E*/*∂β*_*j*_ = ∑_*u*,*v*_(*δ*_*j*_^*l*^∘*d*_*j*_^*l*^)_*uv*_.

### 2.3. Evaluation Indicator

In this paper, six evaluation indexes are selected to measure the experimental results: accuracy, sensitivity, specificity, Matthews correlation coefficient (MCC), *F*_1_ score [13], and training time, and they are calculated by true positive (TP), false positive (FP), true negative (TN), and false negative (NN). Besides, TP indicates that the normal image is predicted to be normal, FP indicates that the abnormal image is predicted to be normal, TN indicates that lung tumor images are predicted as lung tumor images, and FN indicates that the normal lung image is predicted to be abnormal.

(1) Training time: it is the time that the algorithm spends from start to finish; in the process of convolution operation, the total time of the whole training process and testing process is expressed when the specified iteration times are reached
(2) Accuracy: it is the description of the correct classification of the lung CT image; the value is between 0 and 1; the greater the value, the better the classifier; and this value reflects the performance of the correct identification
  (11) $$\begin{array}{c} \text{Accuracy}=\frac{\text{TP}+\text{TN}}{\text{TP}+\text{TN}+\text{FP}+\text{FN}}. \end{array}$$
(3) Sensitivity and specificity: sensitivity indicates that the proportion of the normal lung image is accurately recognized, and specificity indicates that the proportion of lung tumor images is accurately identified:
  (12) $$\begin{array}{c} \text{sensitivity}=\frac{\text{TP}}{\text{TP}+\text{FN}},\\ \text{specificity}=\frac{\text{TN}}{\text{TN}+\text{FP}}. \end{array}$$
(4) MCC: it is a more balanced evaluation standard, which takes into account the true false positives and false negatives; especially, in the case of different numbers, it is generally considered to be a balanced measure. MCC is essentially a correlation coefficient between the observed and predicted binary classifiers and returns the value between -1 and 1, where 1 represents a perfect prediction, 0 represents a random prediction, and -1 indicates that the classification result is completely wrong. The MCC formula is as follows:
  (13) $$\begin{array}{c} \text{MCC}=\frac{\text{TP}\times \text{TN}-\text{FP}\times \text{FN}}{\sqrt{\left(\text{TP}+\text{FP}\right)\left(\text{TP}+\text{FN}\right)\left(\text{TN}+\text{FP}\right)\left(\text{TN}+\text{FN}\right)}}. \end{array}$$
(5) *F*_1_ score: it is an index measuring the recognition performance of two classification models, taking into account the classification accuracy and recall rate, which can be regarded as a kind of weighted average with precision and recall. Its maximum value is 1, the minimum value is 0, and the value that is closer to 1 indicates that the accuracy is higher. The formula is as follows:
  (14) $$\begin{array}{c} \text{Precision}=\frac{\text{TP}}{\text{TP}+\text{FP}},\\ \text{Recall}=\frac{\text{TP}}{\text{TP}+\text{FN}},\\ \text{NegPrecision}=\frac{\text{TN}}{\text{FN}+\text{TN}},\\ \text{NegRecall}=\frac{\text{TN}}{\text{TN}+\text{FP}},\\ F_{1}\text{ score}=\frac{\text{Precision}*\text{Recall}}{\text{Precision}+\text{Recall}}+\frac{\text{NegPrecision}*\text{NegRecall}}{\text{NegPrecision}+\text{NegRecall}}. \end{array}$$

### 2.4. Computer-Aided Diagnosis Model of a Lung Tumor Based on DCNN and Global Fuzzy Feature

#### 2.4.1. Algorithm Idea

In order to verify the feasibility of the convolutional neural network used on medical diagnosis of a lung tumor, a deep convolutional neural network is designed for lung tumor recognition based on the global features of CT images. Although the deep learning model has good generalization ability and robustness [19], the performance of different models is different for different image recognition. According to the fuzzy characteristics of lung CT images and the complexity of medical images, three aspects model parameters, model structure, and optimization algorithm are discussed. Firstly, the effects of different resolution and iteration times on the identification results are discussed. Secondly, different DCNN models are constructed and analyzed from the different convolution kernel sizes, feature dimensions, and network layers. Finally, the different optimization methods on how to influence the DCNN performance from three aspects including sampling methods (maximum pooling and mean pooling), activation function (sigmoid and ReLU), and training algorithm (batch gradient descent and gradient descent with momentum) were discussed. In a word, the research objective is to explore the optimal DCNN model for lung tumor computer-aided diagnosis.

The specific steps for computer-aided diagnosis research of a lung tumor based on DCNN and global features are as follows:

1. Data collection: 5000 CT images with DICOM format were collected from the General Hospital of Ningxia Medical University. The 2500 images were selected as the lung tumor images according to the doctor's mark and the doctor's advice, and the 2500 normal CT images were taken as contrast images
2. Image preprocessing: the collected images were converted into gray image and normalized to the same size of experimental data, and then, lung tumor CT data set was constructed for DCNN training and testing
3. Construction of DCNN: construct a deep convolutional neural network with eight layers aimed at global features of lung cancer, including an input layer, 3 convolutional layers, 3 pooling layers, 2 full connection layers, and an output layer with a softmax classifier
4. Discussion based on different model parameters with the same model structure: the influence of different resolution and the number of iterations on the DCNN recognition rate and training time is discussed aimed at the CT global feature set
5. Discussion of different model structures: based on the initial construction of DCNN, the recognition performance for the lung tumor by changing the convolution kernel size and the number of feature maps and network layers is discussed
6. Comparison analysis of different optimization algorithms: after choosing the suitable model, comparative experiments with different methods were done, including pooling method (mean pooling and maximum pooling), activation function (sigmoid function and ReLU function), and training algorithm (batch gradient descent method and gradient descent method with momentum)
7. Evaluation: Construct an optimal DCNN model through the analysis of experiment and comparison of different model parameters and structures, and it is used on computer-aided diagnosis of a lung tumor based on global features, in order to improve the recognition rate, reduce the training time, and enhance the robustness and generalization ability

#### 2.4.2. Model Construction

**Figure 1:**
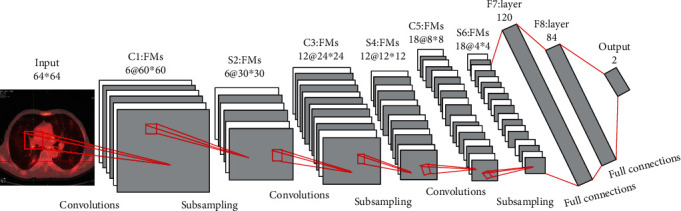
DCNN model construction.

The convolutional neural network can directly input the original image and has obvious advantage to the complex image recognition; besides, CT is widely used in the diagnosis of a lung tumor, but the lesion area accounted for a smaller region in the whole CT images, and the characteristic is not obvious and it is difficult to distinguish, so a deep convolution neural network is constructed to extract the hidden features of the lung tumor for computer-aided diagnosis. The DCNN model structure is shown in Figure 1.

(1) Input layer: the input image is the whole CT image of 64∗64, and it is also the lung global feature input for DCNN classification
(2) C1 layer: it is the first convolutional layer, each neuron is convoluted with the local receptive field of the input image 5∗5, feature maps' size is 60∗60, and it contains 6 different feature maps
(3) S2 layer: it is the first pooling layer, the pooling method was used on 2∗2 neighborhood, feature maps' size is 30∗30, and it contains 12 different feature maps
(4) C3 layer: it is the second convolutional layer, 12 convolution kernels of 7∗7 are used for convolution operation, feature maps' size is 24∗24 after convolution, and it contains 12 different feature maps
(5) S4 layer: it is the second pooling layer, and it got 12 feature maps of 12∗12 after subsampling without repetition
(6) C5 layer: it is the third convolutional layer, and it contains 18 feature maps of 8∗8
(7) S6 layer: it is the third pooling layer, and it contains 18 feature maps of 4∗4
(8) F7 layer: it is the first full connection layer, and it contains 120 neurons and connects with the S6 layer
(9) F8 layer: it is the second full connection layer, and it contains 84 neurons and connected with the upper layer and the output layer
(10) Output layer: it connects with the softmax classifier that is used to calculate the probability of different images that belong to which types. The formula is as follows:
  (15) $$\begin{array}{c} d_{j}^{\left(i\right)}=\frac{\exp\left(W_{j}^{T}x^{\left(i\right)}+a_{j}\right)}{\sum_{j=1}^{2}\exp\left(W_{j}^{T}x^{\left(i\right)}+a_{j}\right)}, \end{array}$$
  where *W* = [*W*_1_, *W*_2_] ∈ *R*^*d*×2^ and *a* = [*a*_1_, *a*_2_] ∈ *R*^*d*×2^ are classifier parameters and *d*_*j*_^(*i*)^ is a possibility prediction for which *x*^(*i*)^ belongs to class *j*; finally, the output is of two types: normal and abnormal lung images.

## 3. Discussion and Conclusion

### 3.1. Experiment Platform

The software and hardware environment is as follows:

Software environment: Windows 7 operating system, MATLAB R2014b

Hardware environment: Intel Xeon CPU E5-2407 v2 @ 2.40 GHz, 32.0 GB memory, 3.5 TB HD

### 3.2. Experimental Data

#### 3.2.1. Data Sources

**Figure 2:**
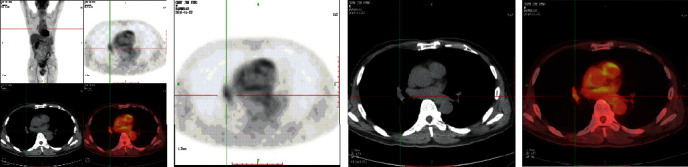
Original experimental data.

5000 CT images were collected from a hospital in Ningxia of China: the 2500 images were selected as the lung tumor images according to the doctor's mark and the doctor's advice, and the 2500 normal CT images were taken as contrast images; the original image is shown in Figure 2.

#### 3.2.2. Data Preprocessing

**Figure 3:**
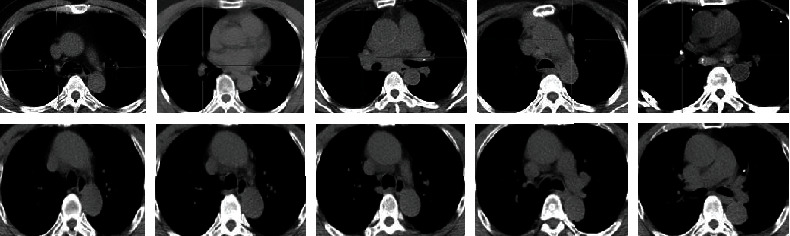
Experimental data after pretreatment.

Firstly, the CT image of a lung tumor was selected according to the marker of three PET/CT modality images; secondly, the normal lung CT image was selected according to the DICOM documents and the doctor's advice; then, the experimental data is converted into a gray image; finally, the experimental data are normalized to the same size: 4000 cases are selected as training data randomly and 1000 cases are regarded as test data. The pretreated images are shown partly in Figure 3: abnormal images are shown in the first line and normal images are shown in the second line.

### 3.3. Analysis of Experimental Results

#### 3.3.1. Experiment 1: Research on Different Model Parameters Based on the Same Model Structure

**Figure 4:**
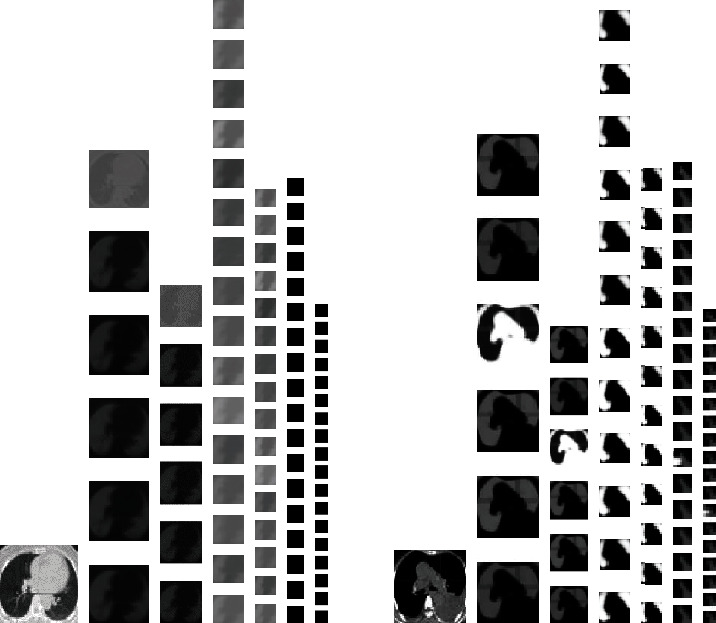
Output of the intermediate feature maps.

*(1) Output of Intermediate Feature Maps*. Image recognition using the deep convolution neural network is based on the abstract features of the hidden layer, and three convolutional layers and three pooling layers are used to extract and output features from different angles after input original images. The output of the intermediate feature maps is shown in Figure 4. From left to right, the feature maps are shown of which the original input image is C1, S2, C3, S4, C5, and S6. It clearly showed that the edge information and contour information of the input image are extracted by the first two layers; that is, the characteristics of low level, such as image edges, lines, and angles, are extracted by the bottom of the convolutional layers. And the abstraction of higher semantic information and essential information is performed by back layers. It cannot be identified with the naked eye, and it has also shown the superior learning ability of deep learning. In a word, the bottom layer of DCNN can learn the physical features such as edge and shape. With the increase in the number of hidden layers in the network, more complex and abstract visual features can be learned.

*(2) The Influence of Different Resolution on Recognition Results*. Due to the different resolution of the images used in the training samples, different convolution and downsampling operations will affect the recognition rate of the model, so based on the same model structure of the convolutional neural network, it was selected among different lung CT images with different resolution for the experiments, including 28∗28, 32∗32, 64∗64, and 100∗100 different resolution images. The experimental results are shown in Table 1.

According to the table, we can see that (1) the higher the resolution of the image, the longer the training time is; that is, the more complex the image is, the longer the training time is. (2) The higher the resolution, the higher the recognition rate, because of the low resolution of the image which means that the input information of the image is lost in different degrees. (3) The sensitivity is generally higher than the specificity regardless of the resolution. The results show that the lung tumor image is easy to be recognized as a normal image, and it also accords with the current situation of pulmonary nodules missed. (4) As for the MCC and *F*_1_ score, the higher the resolution, the higher the value.

In short, high-resolution images will not only lead to more processing time but also reduce the quality of spatial resolution, but the deep convolutional neural network got high accuracy for high-resolution image recognition, so the CT images of 64∗64 resolution are chosen for subsequent experiments that are about different model structures and optimization methods based on the consideration of time complexity and accuracy.

*(3) The Influence of Iterations on Recognition Results*. The iterative method is used to calculate the weight of the convolutional neural network model, the weight and the error will be adjusted in each iteration, and the experimental results will be different with different iterations; in this experiment, the ideal weight parameters are obtained by many iterations, and then, the influence of the number of iterations on the recognition results is discussed. The experimental results are shown in Table 2.

It can be seen from Table 2 that, with the increase in the number of iterations, the accuracy increases first and then decreases, while the training time increases with the iterations increasing. The main reason lies in the idea that the learning of the convolutional neural network is not sufficient when the number of iterations is lower than the normal times. With the increase in the number of iterations, the network has achieved a high recognition rate in the training and learning processes. However, when the number of iterations increases to a certain degree, the recognition rate will decrease. It shows that the network model was trained under the appropriate iterations, the parameters have been optimized to the optimal state, the network also entered the convergence phase, and the network model got the best performance. The increase in the number of iterations will affect the change of the training time, and this change has a positive correlation, and the test time and the number of iterations are not directly linked.

#### 3.3.2. Experiment 2: Recognition Based on Different Model Structures

The deep convolutional neural network is constructed for the first time, and it contains 1 input layer, 3 convolutional layers, 3 pooling layers, 2 full connection layers, and 1 output layer. Input images are lung CT images of 64∗64; the number of feature maps of three convolutional layers are, respectively, 6, 12, and 18; the convolution kernel size is 5∗5, 7∗7, and 5∗5; sigmoid function is regarded as activation function; the output layer connected with the softmax classifier, and the outputs are of two types: normal and abnormal lung images. On the basis of the construction of the deep convolution neural network structure, the convolution kernel size, the number of feature maps, and the depth of the network are changed, and they are used to do further experiments.

*(1) Different Convolution Kernel Sizes*. In order to discuss the influence of different convolution kernel sizes on DCNN recognition results, the deep convolutional neural network structure was fixed and the DCNN was trained by using different convolution kernels; the results are shown in Table 3.

At first, the three convolution kernels of 5∗5, 7∗7, and 5∗5 are adopted, and the recognition rate was 85.3%. Then, the convolution kernel size was reduced to 5∗5, and the recognition rate reduced to 69.7%. Next, the recognition rate reached 80.9% when the convolution kernel sizes were 5∗5, 9∗9, and 9∗9. When the recognition rate reached 86.3%, the convolution kernel sizes were 5∗5, 11∗11, and 11∗11; however, when the convolution kernel sizes increase to 11∗11, 11∗11, and 9∗9, the recognition rate decreased. In a word, with the increase in convolution kernel size, the running time increases; the smaller the convolution kernel size, the less the training time, because small convolution kernels have less training parameters and the space complexity and time complexity are reduced. However, when the convolution kernel is too large or too small, the recognition rate will be reduced. When the convolution kernel is too small, it cannot extract the valid local features; when the convolution kernel is too large, the complexity of the extracted feature may be difficult to express by the convolution kernel. In general, small convolution kernel can handle images finely, but it needs more layers to achieve good results; the large convolution 5kernel can extract abstract features of images, but it needs more training parameters.

When the convolution kernels are 5∗5, 11∗11, and 11∗11, the sensitivity is 99.6% and the sensitivity is higher than the specificity. The MCC and *F*_1_ score are consistent with the recognition rate, and the *F*_1_ score reached 0.86 with the choice of the optimal convolution kernel. Therefore, the convolution kernel size should be set reasonably combined with the input image size, and it is very important to improve the performance of CNN but also to protect the CNN parameter tuning. After the discussion of different convolution kernel sizes, it chooses the convolution kernel of 5∗5, 11∗11, and 11∗11 and carries on the following experimental analysis under the high recognition rate.

*(2) Different Feature Maps*. The number of feature maps is the number of features extracted from each layer, with the same number of convolution kernels per layer. On the premise that the convolution kernel size is invariable, in this paper, we change the number of feature maps on the basis of 6-12-18 and discuss the influence of the extracted feature dimensions on the recognition results; the experimental results are shown in Table 4.

As we can see from Table 4, the number of feature maps is reduced and the running time is reduced, but the recognition rate is not significantly increased. With the increase in the number of feature maps of the third layer, the running time is obviously rising and the recognition rate is also increasing. Although the number of feature maps continues to increase, the number of CT image features is more and the training time is also increasing, and the recognition rate and other evaluation indexes are decreased; especially, the number of features achieves 16-32-200; it took more than two hours, but the recognition rate was only 71.7%. On the whole, with the increase in the number of extracted features, the recognition rate, sensitivity, specificity, MCC, and *F*_1_ score were all increased; when the number of features is 6-12-24, the recognition rate reaches 89.3%. The experimental results show that the number of feature maps of the first layer is less, the number of features in the back layer was increased by 2 times, and it can achieve the highest recognition rate.

Because of the small number of features, the feature description is not sufficient and the large number of feature maps will be overfitting; therefore, we should refer to the size of the data and complexity of the actual sample to select the number of feature maps or convolution kernels and adjust the feature dimension. Generally, using more convolution kernel will get better performance, and the appropriate features maps will certainly be helpful to achieve the desired classification results. Through the discussion of the number of different feature maps, this paper selects the number of feature maps with 6-12-24 and then carries on the following experiment on the premise of ensuring the high recognition rate and the appropriate running time.

*(3) Different Network Layers*. Although the most essential difference between deep and shallow learning is the number of hidden layers, generally, the more the hidden layers are, the easier it is to learn the underlying features of the image. Aimed at lung CT global features of 64∗64 size, the deep convolutional neural network is constructed, and the influence of the DCNN model on the recognition results is discussed by changing the network layer in this experiment. The results of the specific layer assignment and experiment are shown in Table 5; C1 is the first convolutional layer, S1 is the first pooling layer, C2 is the second convolutional layer, and so on.

As we can see from Table 5, with the increase in the number of network layers, from 2 to 8 layers, the recognition rate increased first and then decreased. The recognition rate has only 50% probability with only one convolutional layer and a pooling layer, and it showed that too small number of layers exerts a tremendous influence on recognition result. As the number of layers increases to 6, the recognition rate is up to 89.3%, and then, by adding a convolutional layer, the recognition rate began to decline to 85.4%; the recognition rate is reduced to 76% when constructing the hidden layer of the 8 layers. In general, the deep network structure can promote the reuse of features and get more abstract features with high-level expression; with the increase in network layers, the recognition rate is also increasing; however, there are too many layers in the network, which requires the much convolution and downsampling operations, and the parameters are increasing. In short, the increasing network layers appropriately will ensure the less running time and the higher recognition rate, but too many layers will lead to excess parameters, and the phenomenon of overfitting occurs and the recognition rate reduced. The two indicators MCC and *F*_1_ score have the same change trend with the recognition rate. When the number of hidden layers is 6, the maximum value of the two indicators shows that the recognition efficiency and the fitting effect of the network structure are the best.

#### 3.3.3. Experiment 3: Recognition Based on Different Optimization Methods

Based on the study of the DCNN model structure and model parameters, the optimization of the DCNN model structure for different optimization methods was discussed. Firstly, two kinds of pooling method were analyzed; then, the two activation functions ReLU and sigmoid were analyzed, and finally, two optimization training methods that are the batch gradient descent method and gradient descent method with momentum are compared.

*(1) Mean Pooling and Maximum Pooling*. The deep convolutional neural network model is composed of two kinds of special hidden layers: the convolutional layer and the pooling layer; however, the pooling layer can reduce the feature dimension, reduce the amount of computation, prevent overfitting, and provide a certain degree of translation and rotation invariance. At present, the commonly used sampling methods are mean pooling and max pooling [20]: mean pooling is the average of the feature points in the neighborhood and max pooling is the maximum value of the feature points in the neighborhood. Two groups of experiments were conducted to discuss the effects of different sampling methods on the final results, the learning rate is 0.0005, and the batch size is 200; the experimental analysis was carried out using two DCNN, and the results are shown in Tables 6 and 7.

The results show that the recognition rate of using the max pooling is higher than that of the mean pooling with the same DCNN model, but the two methods have little effect on the training time. When the number of feature maps is 6-16-120 and the number of iterations is 12, the recognition rate of using the max pooling method is up to 79.94%, but the recognition rate of using the mean pooling method is 76.65%. With the increasing iterations, the recognition rate increased first and declined next based on two methods. When the number of feature maps is reduced to 6-16-24, the convergence rate is slow, and the recognition rate of using max pooling is 86.83% and the recognition rate of using mean pooling is about 83.43%. In conclusion, using max pooling is superior to the mean pooling method for the identification of the CT global features on lung tumors.

According to the theory of pattern recognition, the error of feature extraction mainly comes from two aspects: the first is the increase in the variance of the estimation result caused by the limited size of the neighborhood and the other is the deviation of the estimated mean value caused by the parameter error of the convolutional layer [21]. In general, the mean pooling can reduce the first error and more retain the background information of the image, while the max pooling can reduce the second kinds of error and retain the texture information. In the average sense, it is similar to the mean pooling, and in the local sense, it obeys the principle of max pooling; therefore, we should pay more attention to the ROI of the lung CT image; that is to say, the lesion area is more reserved for the local area; hence, the max pooling is better than the mean pooling for the lung tumor recognition based on global features.

*(2) Sigmoid Function and ReLU Function*. The activation function can be joined by the nonlinear factor, because the expression ability of the linear model is not enough, and the function is mapped to the specified range by the activation function. There are two kinds of activation functions: sigmoid function and ReLU function [22]. Sigmoid function is one of the most commonly used activation functions; the formula is as follows:

(16) $$\begin{array}{c} S\left(z\right)=\frac{1}{1+\exp\left(-z\right)}. \end{array}$$

The calculation of the ReLU activation function can be greatly reduced, and it is helpful to the characteristic effect; the formula is as follows:

(17) $$\begin{array}{c} R\left(z\right)=\max\left(0,x\right). \end{array}$$

Two different DCNN models were selected aimed at different activation functions, and the effects of the two common activation functions on the global feature recognition of lung tumors were discussed on the basis of the max pooling. The results are shown in Table 8.

It can be seen from the table that when the structure of the DCNN model is unchanged, the sigmoid function is used to achieve the recognition rate of 73.82% when the iteration is 150 times, and the recognition rate reached 72.07% by using the ReLU function in the iteration of 3; compared with the saturation activation function, ReLU has a faster convergence rate and lower training error. Although the recognition rate of using ReLU activation function is not significantly higher than that of the sigmoid function, it converges quickly and the training time is significantly reduced, so we can use the ReLU function to speed up the convergence rate, reduce the training time, and improve the recognition performance.

*(3) Batch Gradient Descent Method and Gradient Descent Method with Elastic Momentum*. The batch gradient descent method [23] is used to do iterative training and parameter tuning by selecting different batch sizes; in the experiment, the effect of batch size on classification results was discussed, and the effects of two optimization methods batch gradient descent method and gradient descent method with elastic momentum were compared. The experimental results are shown in Table 9.

As we can see from the table, the batch size is closely related to the identification results; the smaller the batch size, the longer the running time, but the recognition rate will continue to increase; when the batch size is too small, the recognition rate will be maintained at a certain level, because when the batch size is too small or too large, training is not enough and the adjustment of the parameters is not enough, so it makes the recognition rate decreased. Therefore, it is necessary to combine the size of the training set and select the appropriate batch size, in order to ensure each parameter adjustment based on adequate training and backpropagation.

It can be seen from Table 10 that the gradient descent method with the elastic momentum is higher than the batch gradient descent method, its recognition rate achieved 96.4%, the sensitivity and specificity were above 95%, and MCC and *F*_1_ score are close to 1. It is indicated that the gradient descent method with elastic momentum is more suitable for lung CT recognition based on DCNN. The gradient descent method with elastic momentum is used to train the network, which reduces the oscillation of the learning process of the neural network, and the network can converge quickly. It can reduce the sensitivity of the local detailed feature for error surface and suppress the network into local minima effectively.

### 3.4. Conclusions

In the paper, the DCNN is directly used for lung tumor recognition based on CT global features, because of the better feature representation ability of the deep convolutional neural network, and image processing and feature extraction are nonessential. There are three comparison experiments with different model parameters, network structure, and training algorithm; the results verified the feasibility of DCNN for the global characteristics of CT lung tumors, and the experiment results show that the appropriate convolution kernel size, the number of feature maps, and the number of layers of the network can be used to ensure a good recognition performance; being too large or too small will make the feature learning not sufficient for parameter fitting. For lung tumor image recognition, the maximum pooling results are better than the average pooling results; the choice of ReLU activation function can speed up the convergence and reduce the running time; the gradient descent method with momentum not only improves the recognition rate but also makes the recognition rate of DCNN for lung cancer reach 94.6%. Thus, the good feature learning ability and good generalization ability and robustness of the deep convolutional neural network are proven.

In a word, with the deep convolutional neural network, the more the layers, the more the feature maps, the larger the feature space can be represented by the network, and the stronger the feature learning ability of the network. However, the computational complexity is larger, and the phenomenon of overfitting is easy to appear. Therefore, it is necessary to select appropriate layers, the number of feature maps, the convolution kernel size, and other parameters in the practical application of the specific field; in this way, it can train a better model and ensure relatively little training time.

## Figures and Tables

**Table 1:** Effects of different resolution samples on experimental results.

Different resolution	Time (s)	Accuracy (%)	Error (%)	Sensitivity (%)	Specificity (%)	MCC	*F*_1_ Score
28∗28	68.66	76.20	23.80	99.40	53.00	0.59	0.75
32∗32	92.21	68.50	31.50	100.00	37.00	0.47	0.65
64∗64	605.97	87.30	12.70	95.40	79.20	0.76	0.87
100∗100	1328.17	89.70	10.30	99.00	80.40	0.81	0.90

**Table 2:** Effects of different iterations on the experimental results.

Iterations	1	10	30	50	100	150	200	250	300
Accuracy (%)	50.00	59.98	77.25	75.55	83.43	86.83	84.13	83.93	85.33
Time (s)	25.08	256.78	700.35	1250.68	2429.52	3750.23	5016.18	6274.23	7803.60

**Table 3:** Effect of different convolution kernel sizes on experimental results.

Convolution kernel size			Time (s)	Accuracy (%)	Error (%)	Sensitivity (%)	Specificity (%)	MCC	*F*_1_ score
k1	k2	k3							
5	5	5	868.26	69.70	30.30	99.80	39.60	0.49	0.67
5	7	5	779.64	85.30	14.70	80.80	89.80	0.71	0.85
5	9	9	950.25	80.90	19.10	100.00	61.80	0.67	0.80
5	11	11	1150.56	86.30	13.70	99.60	73.00	0.75	0.86
7	5	7	706.79	83.40	16.60	97.60	69.20	0.70	0.83
11	11	9	1765.49	82.30	17.50	99.60	65.40	0.69	0.82

**Table 4:** Effect of feature maps on the experimental results.

Feature maps			Time (s)	Accuracy (%)	Error (%)	Sensitivity (%)	Specificity (%)	MCC	*F*_1_ score
FM1	FM2	FM3							
2	4	8	214.06	86.50	13.50	80.40	92.60	0.74	0.86
3	6	12	443.72	85.40	14.60	81.20	89.60	0.71	0.85
6	12	18	779.64	85.30	14.70	80.80	89.80	0.71	0.85
6	12	24	1161.70	89.30	10.70	92.00	86.60	0.79	0.89
6	12	36	1189.80	86.80	13.20	98.20	75.40	0.76	0.87
6	12	120	1392.19	87.00	13.00	98.60	75.40	0.76	0.87
6	12	150	1463.30	85.10	14.90	78.80	91.40	0.71	0.85
6	12	200	1582.14	86.80	13.20	83.80	89.80	0.74	0.87
6	16	32	2054.51	86.70	13.30	81.60	91.80	0.74	0.87
6	16	120	1812.94	87.90	12.10	93.80	82.00	0.76	0.88
6	16	200	2070.36	85.30	14.70	77.80	92.80	0.71	0.85
16	32	200	8616.49	71.70	28.30	100.00	43.40	0.53	0.69

**Table 5:** Relationship between the number of layers and recognition performance.

Layer	Convolution and pooling layers	Time (s)	Accuracy (%)	Error (%)	Sensitivity (%)	Specificity (%)	MCC	*F*_1_ score
2	C1-S1	117.76	50.00	—	—	—	—	—
4	C1-S1-C2-S2	485.38	82.30	17.50	99.60	65.40	0.69	0.82
5	C1-S1-C2-S2-C3	1161.70	83.80	16.20	72.40	95.20	0.69	0.84
6	C1-S1-C2-S2-C3-S3	1765.49	89.30	10.70	92.00	86.60	0.79	0.89
7	C1-S1-C2-S2-C3-S3-C4	1779.64	85.40	14.60	97.00	73.80	0.73	0.85
8	C1-S1-C2-S2-C3-S3-C4-S4	1881.06	76.00	24.00	65.60	86.40	0.53	0.76

**Table 6:** Experimental results of different sampling methods.

Feature maps	Convolution kernels	Activation function	Pooling	Iterations/accuracy (%)								Time (s)
				5	10	12	15	20	30	50	100	
6-16-120	5-10-10	Sigmoid	Max	60.08	65.37	79.94	73.75	71.76	70.26	69.86	63.67	2738.62
			Avg	50.00	68.66	76.65	68.06	65.37	61.58	57.49	58.48	2718.68

**Table 7:** Experimental results of different sampling methods.

Feature maps	Convolution kernels	Activation function	Pooling	Iterations/accuracy (%)								Time (s)
				50	100	140	150	160	200	250	300	
6-16-24	5-10-10	Sigmoid	Max	75.55	83.34	86.23	86.83	84.63	84.13	83.93	85.33	7524.27
			Avg	69.46	81.74	81.24	83.43	80.54	82.14	83.03	83.83	7620.68

**Table 8:** Experimental results of different activation functions.

Feature maps	Convolution kernels	Pooling	Activation function	Iterations	Accuracy (%)	Time (s)
6-16-120	5-10-10	Max pooling	Sigmoid	150	73.82	543.73
			ReLU	3	72.07	11.37
20-50-500	5-10-10	Max pooling	Sigmoid	8	80.00	4971.42
			ReLU	1	72.82	1043.57

**Table 9:** Effect of batch size on experimental results.

Batch size	Time (s)	Accuracy (%)	Error (%)	Sensitivity (%)	Specificity (%)	MCC	*F*_1_ score
20	1816.22	90.50	9.50	98.80	82.20	0.82	0.90
50	1708.21	91.70	8.30	99.40	84.00	0.84	0.92
100	1619.94	89.90	10.10	97.20	82.60	0.81	0.90
200	1533.37	86.30	13.70	99.60	73.00	0.75	0.86
300	1526.66	85.60	14.40	99.40	71.80	0.74	0.85
500	1508.10	68.40	31.60	100.00	36.8	0.47	0.65

**Table 10:** Results of the batch gradient descent and gradient descent method with elastic momentum.

Training method	Time (s)	Accuracy (%)	Error (%)	Sensitivity (%)	Specificity (%)	MCC	*F*_1_ score
Batch gradient descent	5809.73	91.70	8.30	99.40	84.00	0.84	0.92
Gradient descent method with elastic momentum	5016.18	96.40	3.60	97.60	95.20	0.93	0.96

## Data Availability

Some or all data and models during the study are available from the corresponding author on request.
